# Supplementary material for: Genome-wide association study meta-analysis of blood pressure traits and hypertension in sub-Saharan African populations: an AWI-Gen study
Source: Nat Commun. 2023 Dec 16;14:8376. doi: 10.1038/s41467-023-44079-0 (PMC10725455; doi:10.1038/s41467-023-44079-0)
Supplement: Supplementary file 1 — Supplementary Information [file 41467_2023_44079_MOESM1_ESM.pdf]

SUPPLEMENTARY INFORMATION

Genome-wide Association Study Meta-analysis of Blood Pressure Traits and Hypertension in Sub-Saharan African Populations: An AWI-Gen Study

Singh, S.[1,2*], Choudhury, A. [1], Hazelhurst, S.[1,3], Crowther, N. J.[4], Boua, R. P.[1,5], Sorgho, H.[5], Agongo, G.[6,7], Nonterah, E.A.[7,8], Micklesfield, L. K.[9], Norris, S. A.[9,10], Kisiangani, I.[11], Mohamed, S.[11], Gómez-Olivé, F. X.[12], Tollman, S. M.[12], Choma, S.[13], and Brandenburg, J.T.[1+] Ramsay, M.[1,2+]

[1] Sydney Brenner Institute for Molecular Bioscience, Faculty of Health Sciences, University of the Witwatersrand, Johannesburg, South Africa

2 Division of Human Genetics, School of Pathology, National Health Laboratory Service and Faculty of Health Sciences, University of the Witwatersrand, Johannesburg, South Africa

[3] School of Electrical and Information Engineering, University of the Witwatersrand, Johannesburg, South Africa

[4] Department of Chemical Pathology, National Health Laboratory Service, Faculty of Health Sciences, University of the Witwatersrand, Johannesburg, South Africa

[5] Clinical Research Unit of Nanoro, Institut de Recherche en Sciences de la Sante, Ouagadougou, Burkina Faso

[6] Department of Biochemistry and Forensic Sciences, School of Chemical and Biochemical Sciences, C.K. Tedam University of Technology and Applied Sciences, Navrongo, Ghana.

[7] Navrongo Health Research Centre, Ghana Health Service, Navrongo, Ghana.

[8] Julius Global Health, Julius Centre for Health Sciences and Primary Care, University Medical Centre Utrecht, Utrecht, Netherlands.

[9] SAMRC Developmental Pathways for Health Research Unit, Faculty of Health Sciences, University of the Witwatersrand, Johannesburg, South Africa

[10] School of Health and Human Development, University of Southampton, UK

[11] African Population and Health Research Center, Nairobi, Kenya

[12] "MRC/Wits Rural Public Health and Health Transitions Research Unit (Agincourt), School of Public Health, Faculty of Health Sciences, University of the Witwatersrand, Johannesburg, South Africa"

[13] Department of Medical Science, Public Health and Health Promotion, School of Health Care Sciences, Faculty of Health Sciences, University of Limpopo, Polokwane, South Africa

[+] Equal contributions

*** Corresponding authors:** Surina Singh (surinsingh@hotmail.co.za) and Michele Ramsay (Michele.Ramsay@wits.ac.za).

Supplementary Figures

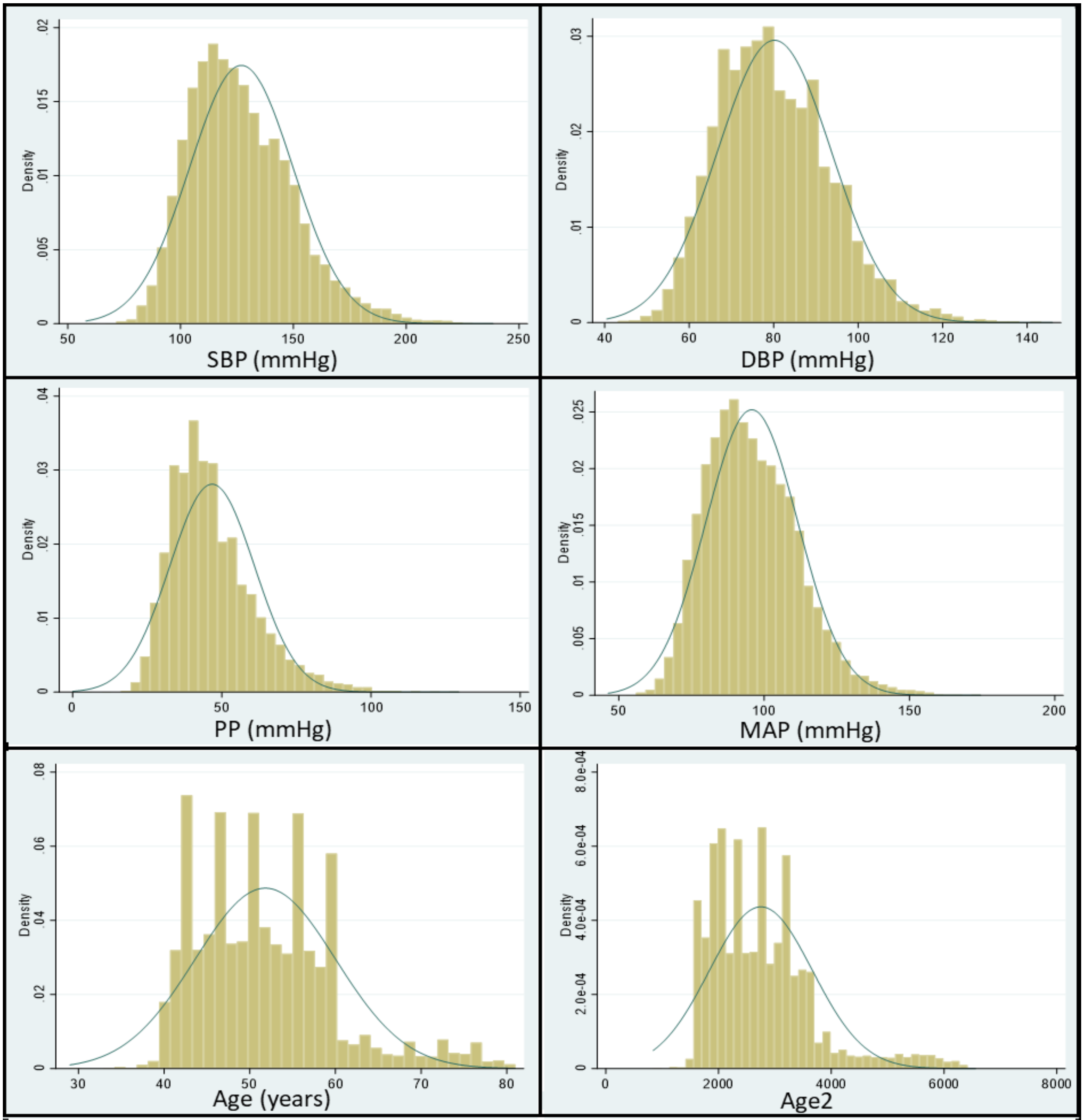

Trait	Mean	SD	6SD of Mean	Min	Min (6SD - Mean)	Min Extreme	Max	Max (6SD + Mean)	Max Extreme	Winso-rised
**SBP**	126,91	22,86	137,16	58	-10,25	FALSE	238,5	264,07	FALSE	None
**DBP**	80,18	13,48	80,88	40,5	-0,7	FALSE	146	161,06	FALSE	None
**PP**	46,74	14,2	85,2	0	-38,46	FALSE	129,5	131,94	FALSE	None
**MAP**	95,75	15,83	94,98	46,33	0,77	FALSE	174,5	190,73	FALSE	None
**Age**	51,82	8,19	49,14	29	2,68	FALSE	81	100,96	FALSE	None
**Age2**	2752,12	913,97	5483,82	841	-2731,7	FALSE	6561	8235,94	FALSE	None

Supplementary Figure 1: Distribution for phenotypes with continuous data.

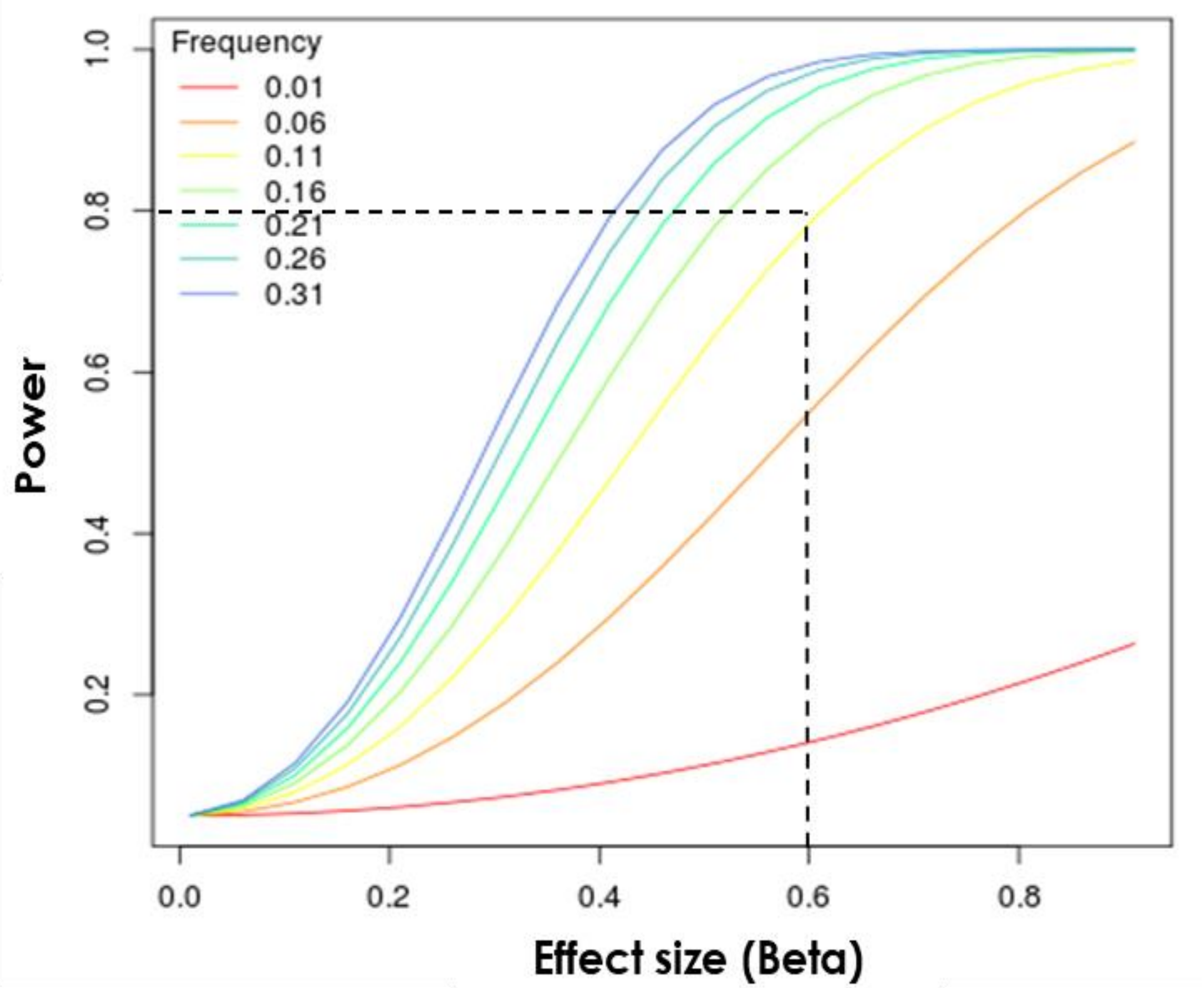

Supplementary Figure 2: Power calculation considering different effect sizes (Beta) and allele frequencies.

Power calculation was conducted using Quanto V1.2.3 [1]. Based on 10,703 individuals for continuous traits. Each coloured line represents a power estimate at a particular minor allele frequency. This revealed that the current study has at least 80% power to detect an effect size beta of ~ 0.60 for SNPs with MAF>0.10.

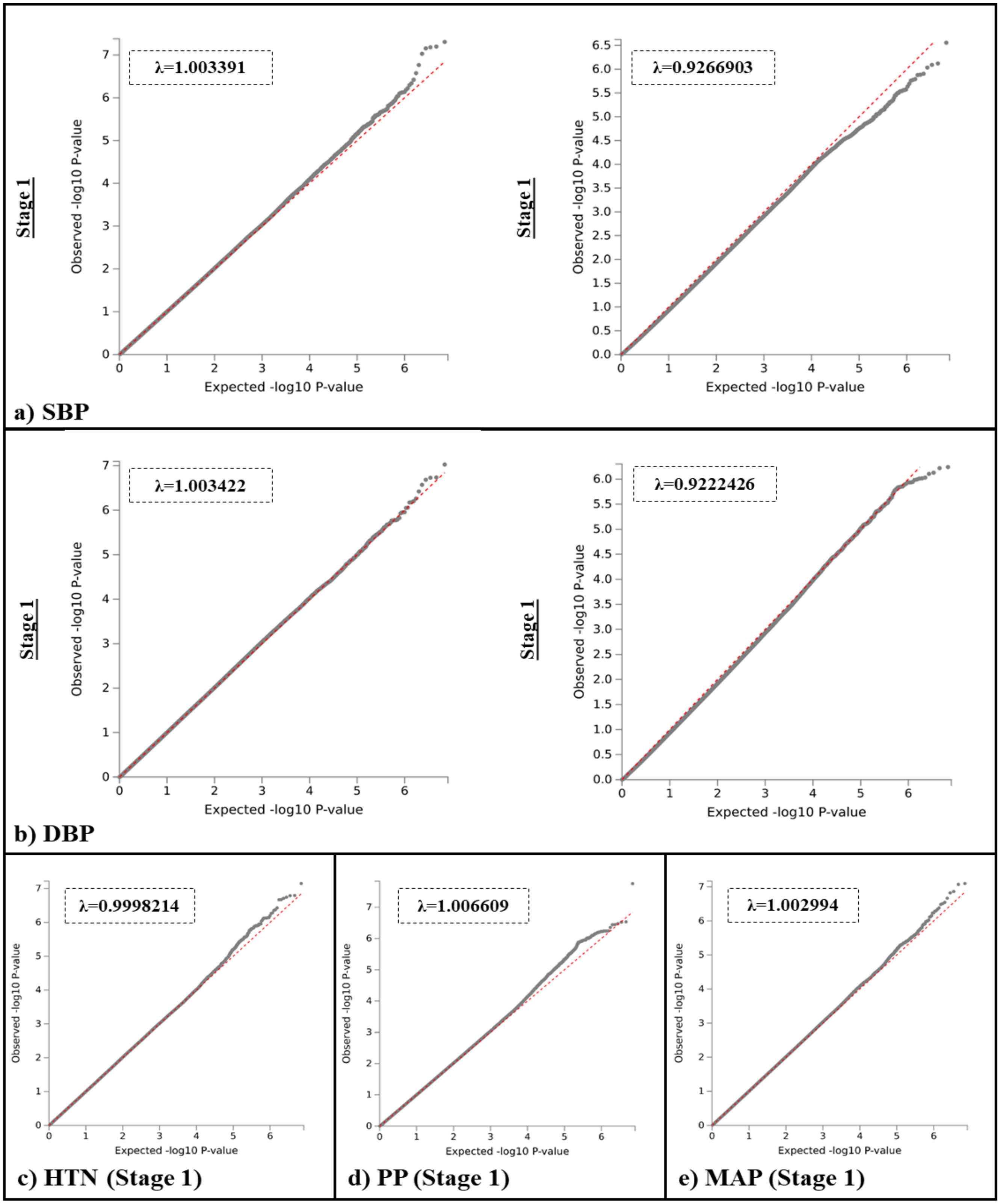

Supplementary Figure 3: Q–Q plots with the genomic control coefficient (λ) showing the discovery GWAS genetic associations in AWI-Gen (Stage 1) and the meta-analysis (Stage 2) for five BP traits.

Adjusting for age, age2, sex and the first 10 PCs as covariates. With GW significance=p<5E-8. QQ-plot shows the distribution of –log10-transformed p-value for observed (y-axis) vs expected (x-axis) with GIF (λ); red line=observed; grey line=expected.

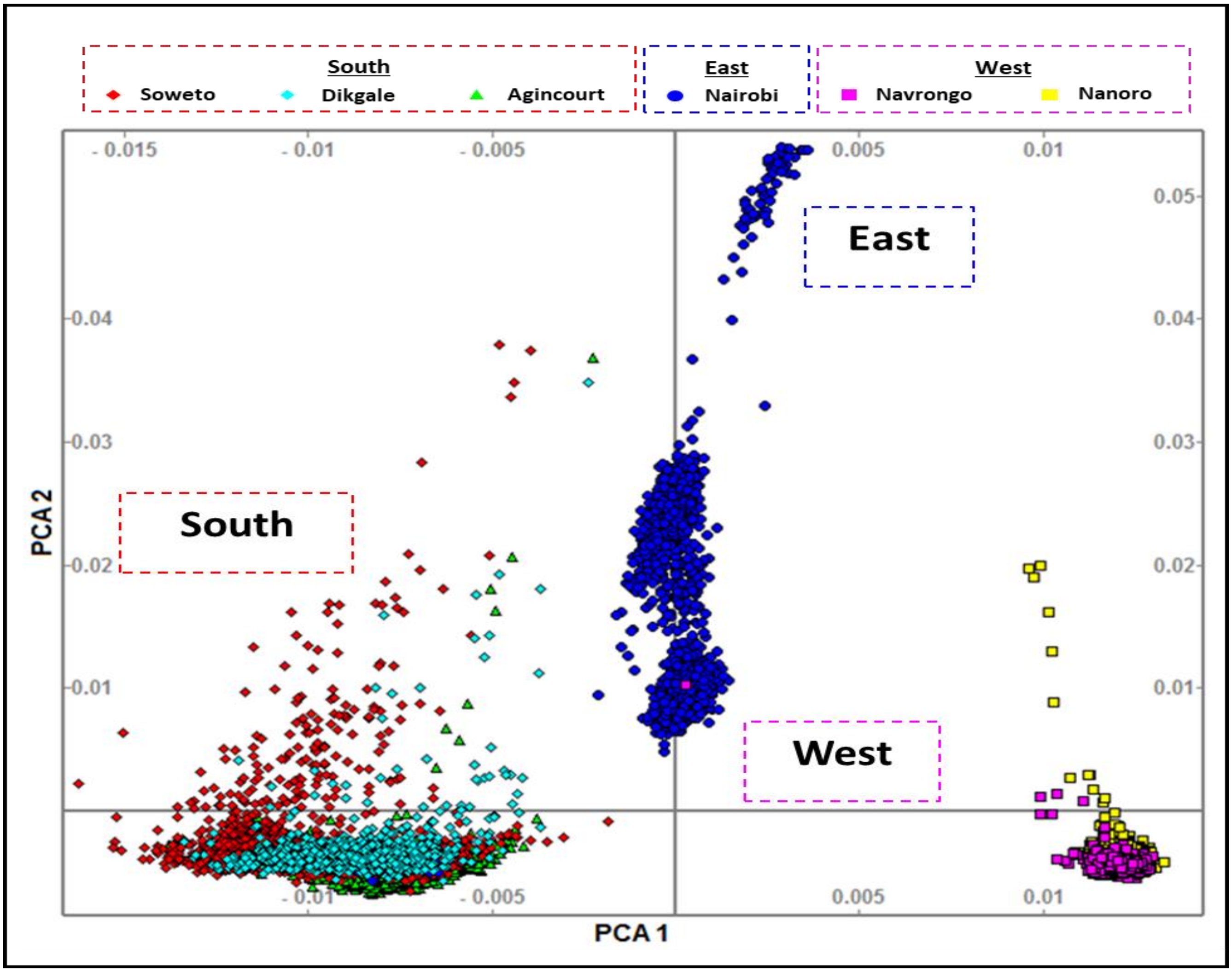

Supplementary Figure 4: Population structure of AWI-Gen across regions, using PCs 1 and 2.

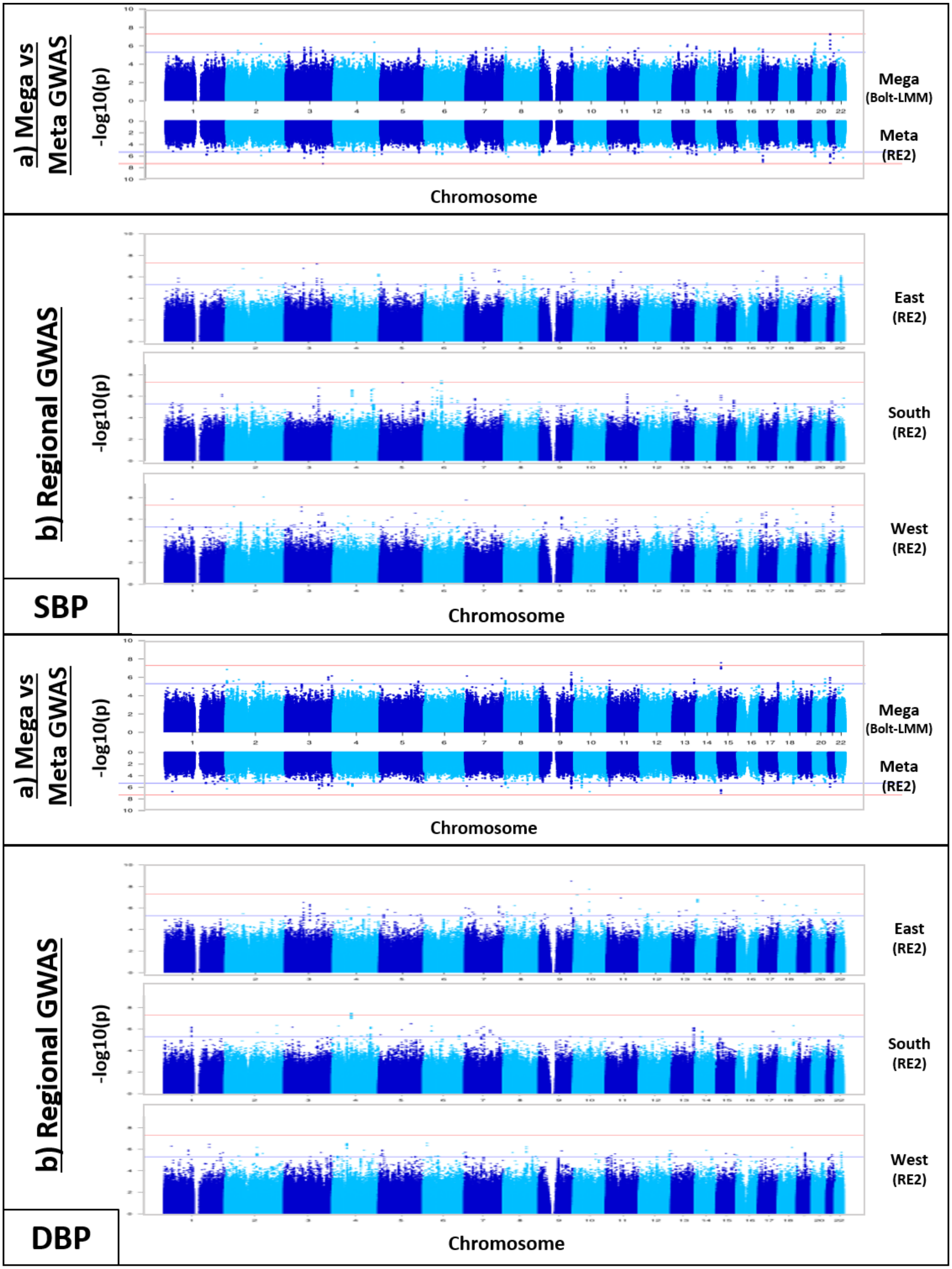
a) Mega vs Meta GWAS
-log10(p)
Mega (Bolt-LMM)
Meta (RE2)
Chromosome
b) Regional GWAS
East (RE2)
South (RE2)
West (RE2)
SBP
a) Mega vs Meta GWAS
Mega (Bolt-LMM)
Meta (RE2)
b) Regional GWAS
East (RE2)
South (RE2)
West (RE2)
DBP

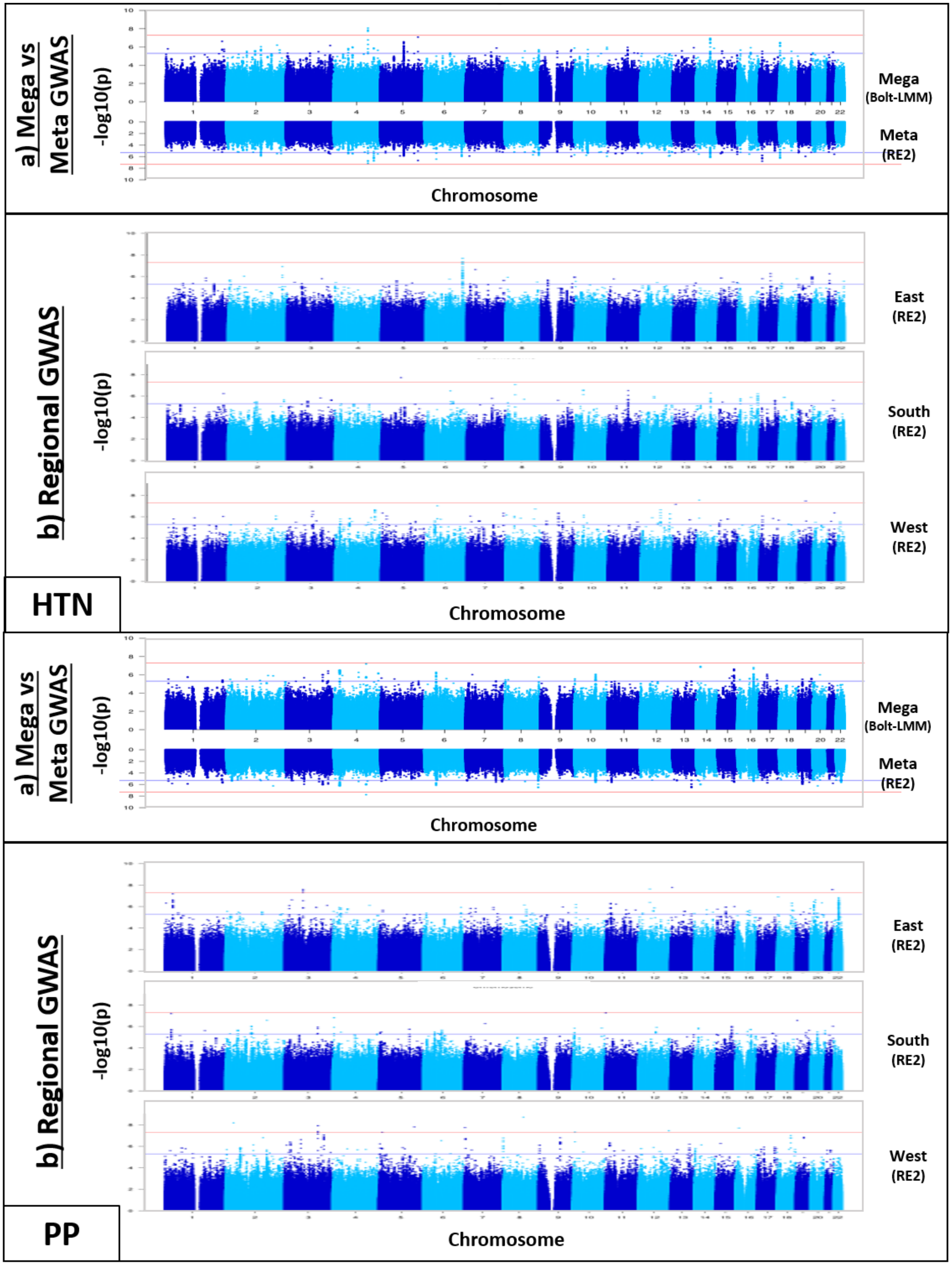
a) Mega vs Meta GWAS
-log10(p)
Mega
(Bolt-LMM)
Meta
(RE2)
Chromosome
b) Regional GWAS
-log10(p)
East
(RE2)
South
(RE2)
West
(RE2)
HTN
Chromosome
a) Mega vs Meta GWAS
-log10(p)
Mega
(Bolt-LMM)
Meta
(RE2)
Chromosome
b) Regional GWAS
-log10(p)
East
(RE2)
South
(RE2)
West
(RE2)
PP
Chromosome

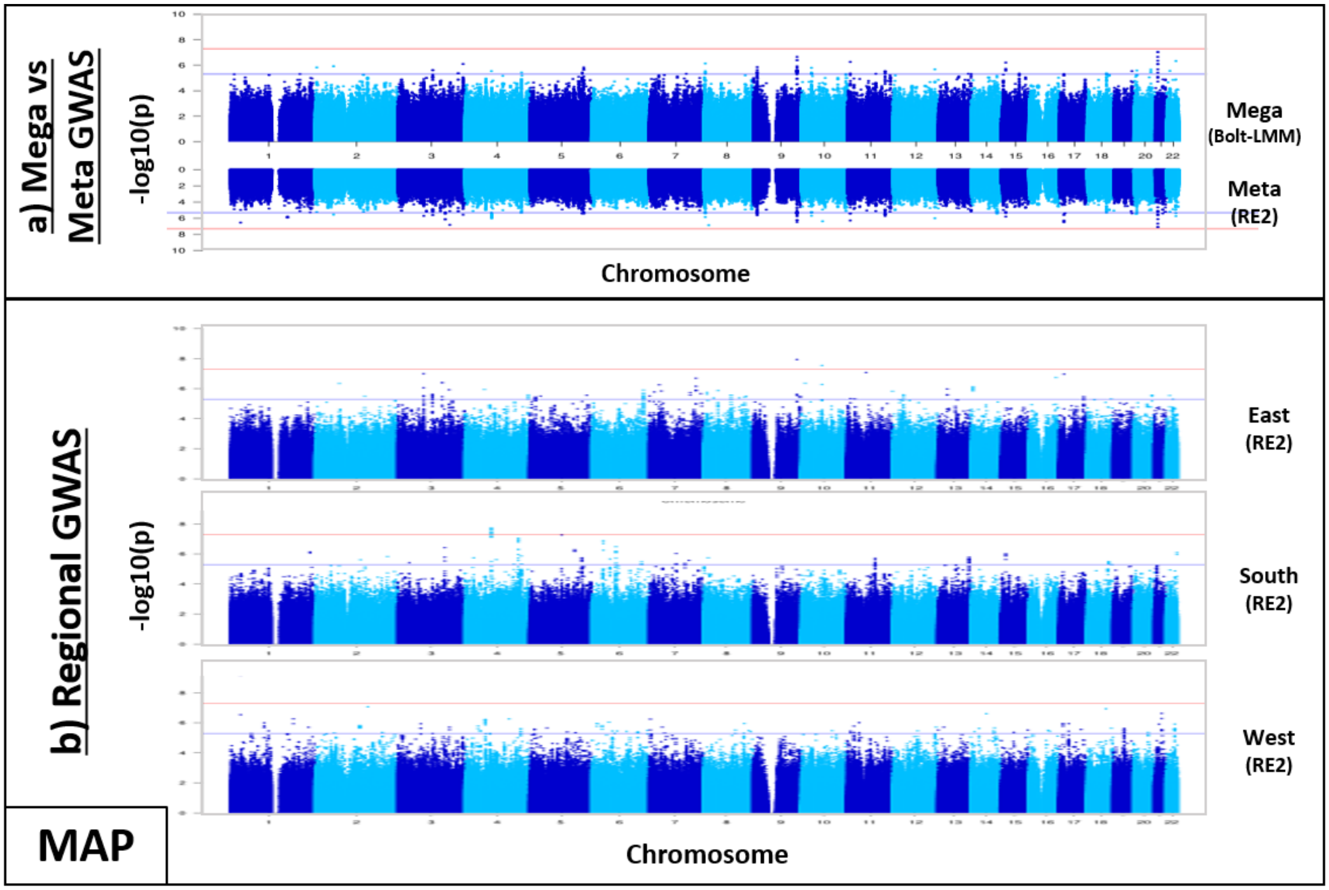

Supplementary Figure 5: AWI-Gen combined mega-analysis vs regional meta-analysis and regional discovery GWAS genetic associations showing Miami plots for five BP traits.
The regional discovery GWAS and mega-analysis were analysed using Bolt-LMM V2.3.2 [2]. The meta-analysis was analysed using RE2 (Han and Eskin's random-effects). Adjusting for age, age2, sex and the first 10 PCs as covariates. With GW significance=p<5E-8. Miami plot shows –log10-transformed two-tailed p-value for each BP trait (y-axis) and base pair positions along the chromosomes (x-axis); red line=Genome-wide significance (p<5E−08); purple line=threshold for suggestive association (p<1E−06).

Pop	AF	P	Mean	Lower	Upper
East	0.79	3.20E-04	-3.21	-4.96	-1.46
South	0.78	6.90E-01	0.21	-0.83	1.25
West	0.83	4.10E-07	-3.12	-4.32	-1.91
Mega (Bolt-LMM)	0.80	7.70E-05	-1.45	-2.17	-0.73
Meta (FE)	0.80	2.58E-05	-1.54	-2.26	-0.82
Meta (RE, using RE2 p-value)	0.80	4.95E-08	-1.99	-4.41	0.44

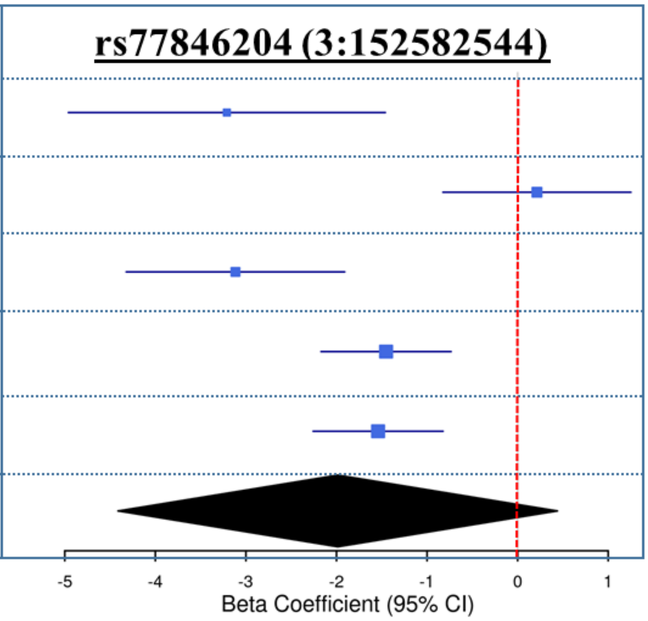

a) SBP (Stage 1)

Pop	AF	P	Mean	Lower	Upper
East	0.98	2.20E-05	-5.68	-8.30	-3.05
South	0.94	9.00E-04	-2.04	-3.24	-0.83
West	0.97	1.70E-03	-2.21	-3.59	-0.83
Mega (Bolt-LMM)	0.96	6.50E-08	-2.36	-3.21	-1.50
Meta (FE)	0.96	1.25E-08	-2.49	-3.35	-1.64
Meta (RE, using RE2 p-value)	0.96	1.76E-08	-2.92	-4.59	-1.25

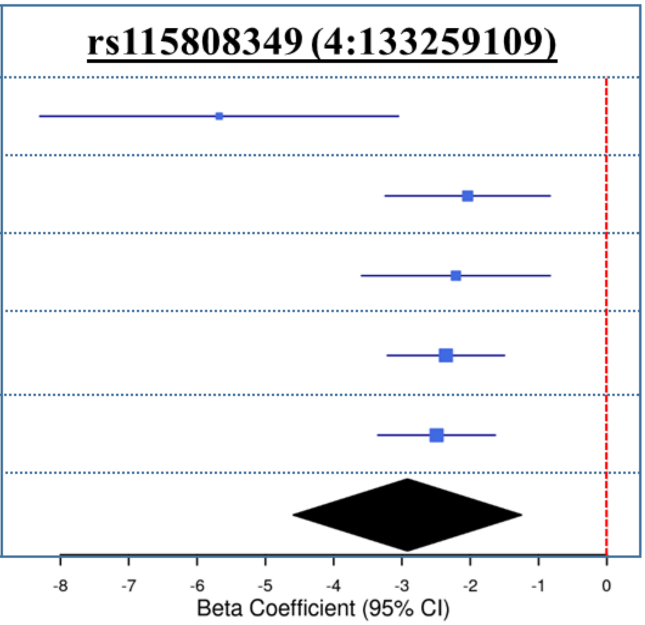

b) PP (Stage 1)

Supplementary Figure 6: Forest plot showing the effect sizes of the novel GW significant associations (p<5E-08) for (a) SBP and (b) PP in the different geographic regions of the AWI-Gen study.

Effect sizes for the novel GW significant SNPs for the AWI-Gen study (combined cohort mega-analysis) and the meta-analysis of the three AWI-Gen region GWASs (Stage 1 analysis – using two different approaches): (a) Plots shown for SBP around the *P2RY1* region (rs77846204, p=4.95E-08) and (b) PP around the *LINC01256* region (rs115808348, p=1.76E-08, intergenic *ELL2P2* – also consisting of rs62317311 (p=8.92E-07), for the AWI-Gen Stage 1 GWAS (N=10,775). The effect size (Beta Coefficient) of each genetic variant and the 95% confidence interval (CI) is represented on the X-axis. When the CI spans 0 (stippled red line), the association is not significant. Significant associations to the left of the line indicate a negative association (i.e. associations with decreased SBP or PP). The diamond-shaped summary statistic represents the overall effect size and confidence interval for the RE2 (Han and Eskin's random-effects) model. Pop=the type of population or analysis used, AF=effect allele, mean=effect size.

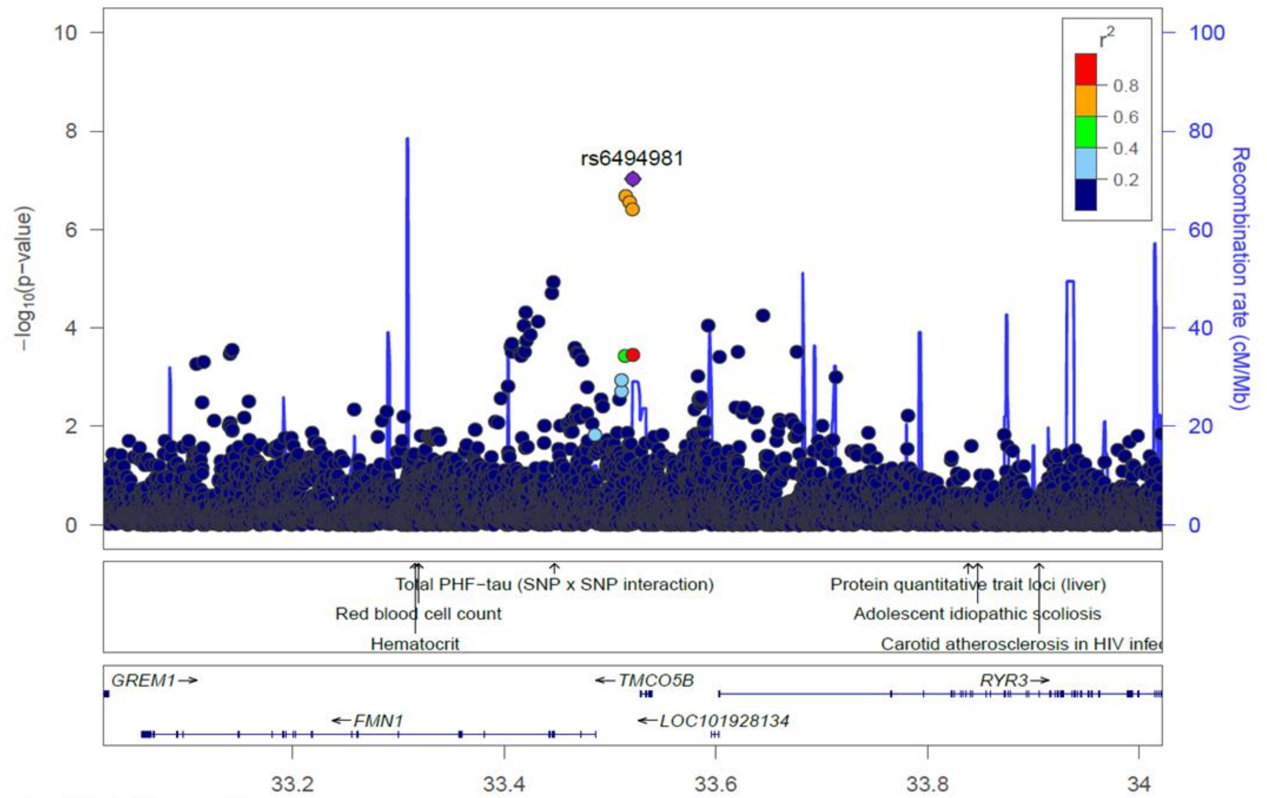

a) DBP (Stage 1)

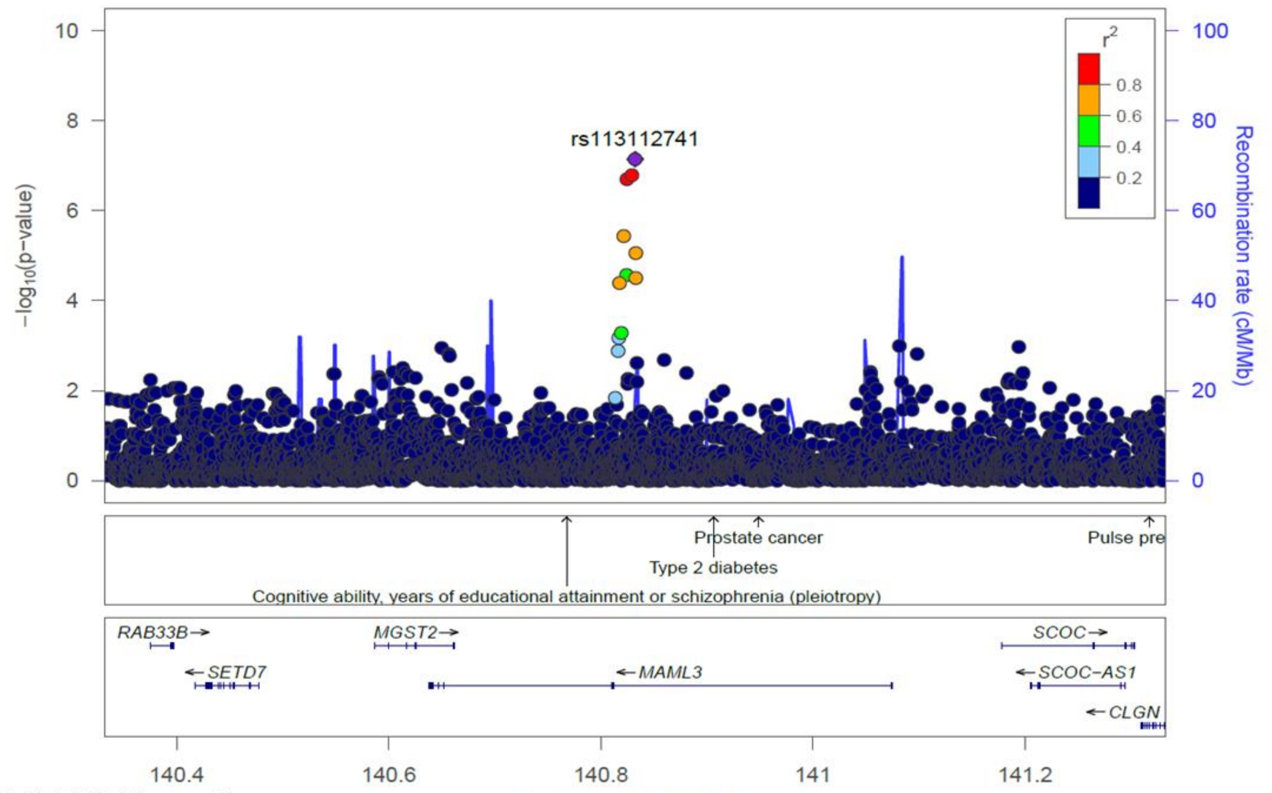

b) HTN (Stage 1)

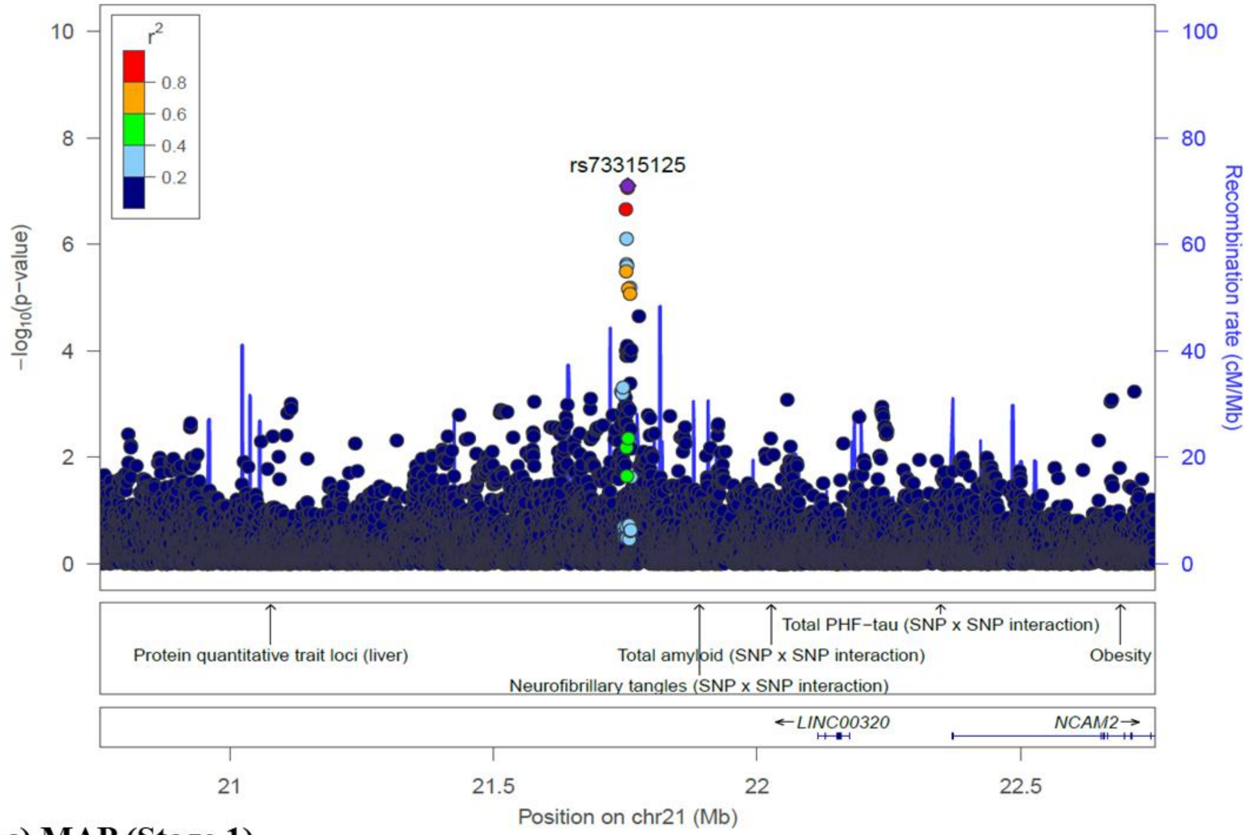

c) MAP (Stage 1)

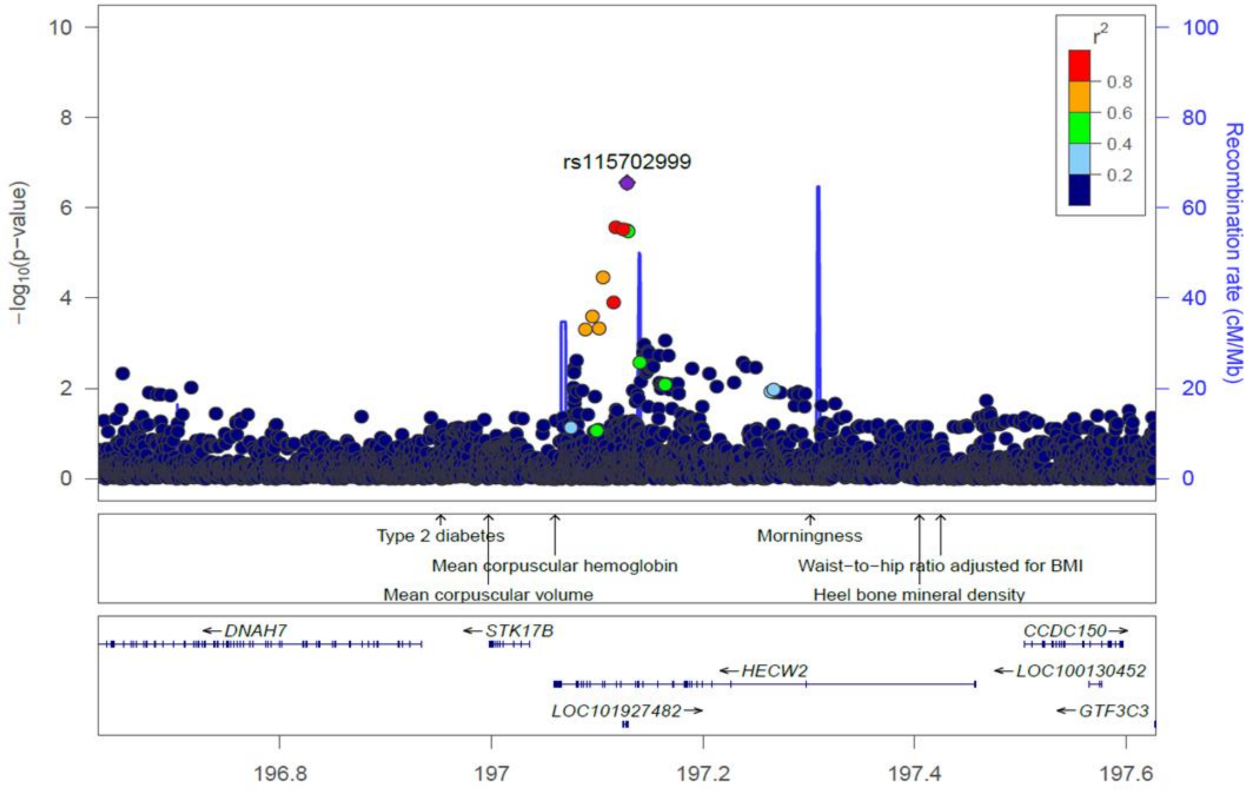

d) SBP (Stage 2)

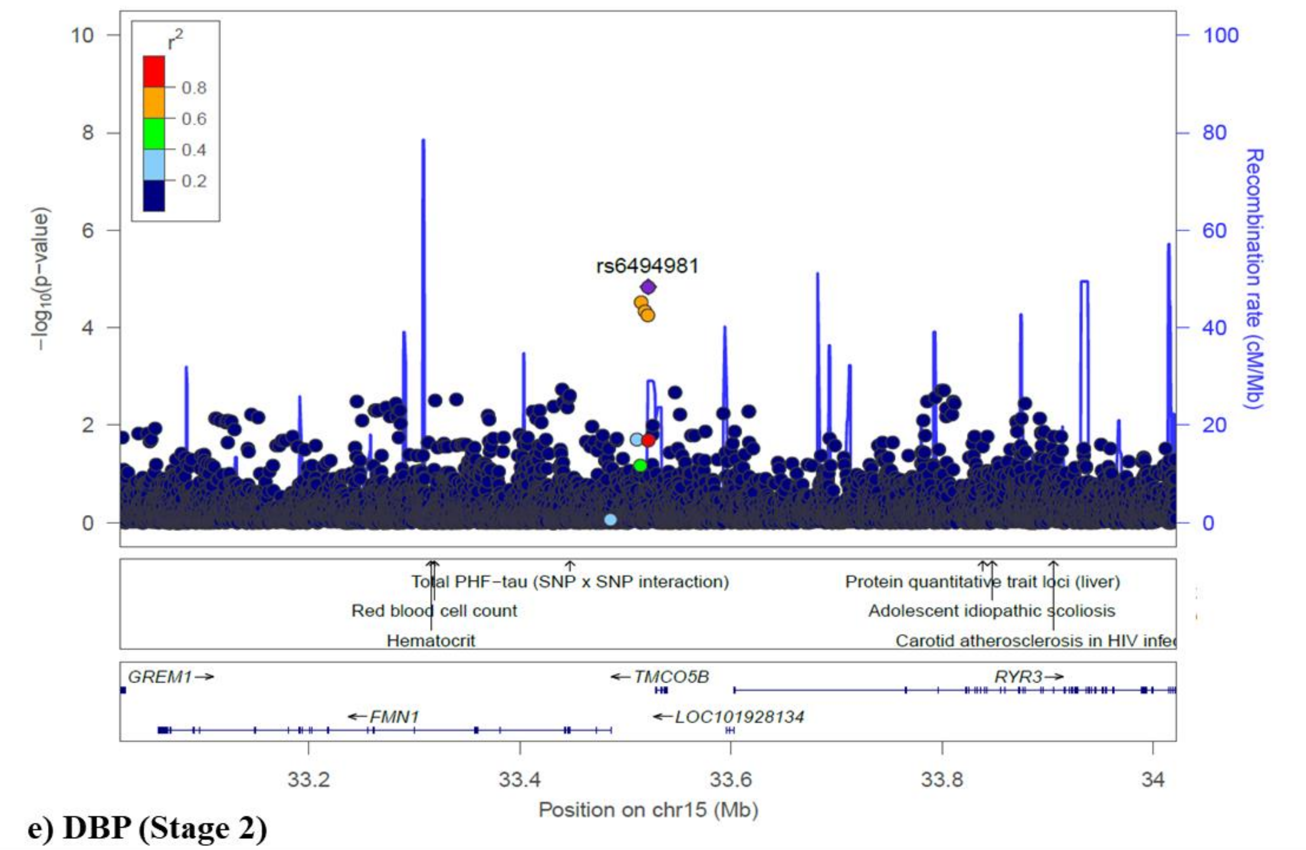

e) DBP (Stage 2)

Supplementary Figure 7: Regional plots of the top suggestive significant (p<5E-06) associations for BP-related traits in the AWI-Gen study for the Stage 1 and 2 GWAS.

Locuszoom (https://my.locuszoom.org/) plots showing associations for GW significant associations. Lead SNPs (purple diamond), GWAS Catalog trait labels and genes are labelled. Plots are shown for (a) DBP around the *TMCO5B* region (rs6494981, p=9.40E-08), (b) HTN around the *MAML3* region (rs113112741, p=7.12E-08) and (c) MAP around the *LINC00320* region (rs73315125, p=8.02E-08, nearing *FDPSP6*) for the AWI-Gen Stage 1 GWAS (N=10,775). Plots are also shown for (d) SBP around the *HECW2* region (rs115702999, p=2.77E-07) and (e) DBP around the same region as Stage 1 i.e. *TMCO5B* region (rs6009081, p=5.75E-07, nearing *PPARA*) for the Stage 2 AWI-Gen meta-analysis GWAS.

Supplementary Notes

The supplementary notes include extra method details not necessary in the main paper.

Supplementary Note 1: QC

The H3ABioNet/H3Agwas QC pipeline workflow [3] (https://github.com/h3abionet/h3agwas/tree/master/qc) was used to conduct QC analysis for the AWI-Gen dataset, as previously described [4]: (1) SNPs with high missingness (>0.02), low minor allele frequency (MAF) (<0.01), and extreme deviation from Hardy Weinberg Equilibrium proportions (HWE) (<0.0005) were excluded; (2) Samples with high genotype missingness (>0.01) and discordant sex information were removed; (3) Mitochondrial, Y and X chromosome SNPs, including SNPs that did not match the Genome Reference Consortium Human Build 37 (GRCh37/hg19) reference alleles were removed. The same steps were followed for the UKBBa dataset.

Supplementary Note 2: Genetic association analysis

1. **Discovery GWAS**
   - **Reason for RE2:** There are major differences in the prevalence of HTN in different regions of the continent. Also, environmental and lifestyle factors, which are key modifiers of the condition, have been reported to differ substantially between the study sites. Therefore, we considered it appropriate to allow for some level of heterogeneity in the meta-analysis and focused on RE2-based P-values. This approach assumes no heterogeneity of effect sizes if the null hypothesis is true (i.e. all beta values are zero), thus correcting for the overly conservative standard RE (random-effects) meta-analysis approach. The RE2 model has been shown to achieve higher statistical power than FE, when there is heterogeneity (unlike the traditional RE), indicating that using RE2 improves detection for discovering associations in GWAS meta-analysis [5]. For comparison, FE (fixed-effects) models, using the same parameters as the RE2 model, were conducted.
   - **FE results compared to RE2:**
     - The result of FE can be found in Table S 6 and Table S 7.
     - SBP significant SNP for the Stage 1 RE2 GWAS (rs77846204, p=4,95E-08) was not GW for the fixed effects (FE) model (p=2.58E-05). Another SNP within the same genomic region was GW significant (rs73315125, intergenic variant in *FDPSP6*, p=4.56E-08), which had suggestive significance when applying the RE2 model (p=6.35E-08) (Table S 4, Table S 6) due to variability of effect between region (Supplementary Figure 6).
     - PP significant SNP for the Stage 1 RE2 GWAS (rs115808349, p=76E-08) also met GW significance for the fixed effects (FE) model (p=1.25E-08) (Table S 6).
2. **Visualization and interpretation of genetic associations:**
   - **Genomic control** was evaluated in R [6], by calculating GIF as: $\lambda = \frac{X^2 median}{0.456}$, i.e. $X^{2=}$chi-squared of observed and 0.456=median chi-squared of expected, where λ=1 means that the population is

homogenous and λ>1.05 means that correction for population structure was not done efficiently and needs to be re-corrected.

- **Q-Q plots** were constructed in FUMA [7] to assess deviation in the distribution of expected and observed p-values.

3. **Replication with previous findings:**

- **The GWAS Catalog database** was downloaded (https://www.ebi.ac.uk/gwas/, accessed on 27 March 2022). Since the genome assembly of the GWAS Catalog was the Genome Reference Consortium Human Build 38 (GRCh38/hg38), it was converted to GRCh37/hg19, to allow for comparison, by conducting a lift-over. A subset of the GWAS Catalog data was generated by filtering for keywords relevant to BP traits: "Systolic blood pressure", "Diastolic blood pressure", "hypertension", "pulse pressure", and "mean-arterial pressure".

4. ***In silico* functional analysis:**

- **Candidate SNPs functional consequences** were predicted by chromosome base-pair position, and reference and alternate alleles, to databases containing known functional annotations. This included: (1) ANNOVAR, a variant annotation tool which is used to obtain functional consequences of SNPs on gene functions [8]; (2) combined annotation-dependent depletion (CADD), a score of deleteriousness of SNPs predicted by 63 functional annotations with a threshold of>>12.37 to be deleterious [9]; (3) RegulomeDB (RDB), a categorical score representing regulatory functionality of SNPs based on expression quantitative trait loci (eQTLs) and chromatin marks [10], with eQTLs scans using the Genotype-Tissue Expression (GTex) Consortium [11].

Supplementary Note 3: PRS

1. **PRSice-2 V2.3.5** [12], a PRS software, was used to calculate and interpret PRSs. These steps included: (1) clumping (LD adjustment) to identify and select the most significant SNP in each LD block for further analyses; (2) p-value threshold (shrinkage strategy) to remove very low or non-significantly associated SNPs by performing multiple PRS analyses with varying p-value, and (3) plotting PRS results (Clumping distance=250kb, R2=0.1, P- value thresholds for 1 to 5E-8).
2. **The P-threshold value (PT),** was determined using PRSice-2, by calculating the empirical P-value i.e. $Empirical - P = \frac{= \sum Nn{=}1 I(Pnull{<}Po){+}1}{N+1}$ (where Po is the best p-value threshold; $Pnull$ is the p-value of association of the best p-value threshold under the null; and I is the indicator function).

References

1 Gauderman, W. & Morrison, J. Quanto 1.2. 3: a computer program for power and sample size calculations for genetic-epidemiology studies. (2007).

2 Loh, P.-R. (2015).

3 Brandenburg, J.-T. *et al.* H3AGWAS: a portable workflow for genome wide association studies. *BMC Bioinformatics* **23**, 1-15 (2022).

4 Choudhury, A. *et al.* Meta-analysis of sub-Saharan African studies provides insights into genetic architecture of lipid traits. *Nature Communications* **13**, 1-13 (2022).

5 Han, B. & Eskin, E. Random-effects model aimed at discovering associations in meta-analysis of genome-wide association studies. *The American Journal of Human Genetics* **88**, 586-598 (2011).

6 Team, R. C. R: A language and environment for statistical computing. (2013).

7 Watanabe, K., Taskesen, E., van Bochoven, A. & Posthuma, D. Functional mapping and annotation of genetic associations with FUMA. *Nature Communications* **8**, 1826, doi:10.1038/s41467-017-01261-5 (2017).

8 Wang, K., Li, M. & Hakonarson, H. ANNOVAR: functional annotation of genetic variants from high-throughput sequencing data. *Nucleic Acids Research* **38**, e164-e164 (2010).

9 Kircher, M. *et al.* A general framework for estimating the relative pathogenicity of human genetic variants. *Nature Genetics* **46**, 310-315 (2014).

10 Boyle, A. P. *et al.* Annotation of functional variation in personal genomes using RegulomeDB. *Genome research* **22**, 1790-1797 (2012).

11 Consortium, G. The GTEx Consortium atlas of genetic regulatory effects across human tissues. *Science* **369**, 1318-1330 (2020).

12 Euesden, J., Lewis, C. M. & O'reilly, P. F. PRSice: polygenic risk score software. *Bioinformatics* **31**, 1466-1468 (2014).
