# Supplementary material for: Genome-wide association study meta-analysis of blood pressure traits and hypertension in sub-Saharan African populations: an AWI-Gen study
Source: Nat Commun. 2023 Dec 16;14:8376. doi: 10.1038/s41467-023-44079-0 (PMC10725455; doi:10.1038/s41467-023-44079-0)
Supplement: Supplementary file 3 — Description of Additional Supplementary Files [file 41467_2023_44079_MOESM3_ESM.pdf]

Description of Additional Supplementary Files

Supplementary Data 1: BP definitions, according to JNC7 guidelines.

BP traits, systolic blood pressure (SBP), diastolic blood pressure (DBP), hypertension (HTN), pulse pressure (PP) and mean-arterial pressure (MAP), measured according to JNC7 guidelines [66].

Supplementary Data 2: Comparison of HTN Status (N=10775).

HTN Status is derived from continuous traits i.e. systolic blood pressure (SBP) and diastolic blood pressure (DBP).

Supplementary Data 3: GW associations of AWI-Gen's combined mega-analysis vs, independent regional discovery at p<5E-08.

Mega-analysis of AWI-GEN regions vs independent regions i.e. East (E), South (S) and West (W) African subsets. Given for Build 37 (GRCh37/hg19).
Arranged in order of trait, Chr and position and SNP. 437 SNPs with duplicate SNPs and positions highlighted in bold and red respectively.
Abbreviations: CHR, chromosomes; POS, gene position; SNP, single nucleotide polymorphism; EA, effect allele; RA, reference allele; EAF, effect allele frequency; Beta, effect size estimates for continuous trait i.e. systolic blood pressure (SBP), diastolic blood pressure (DBP), pulse pressure (PP) and mean-arterial pressure (MAP); OR, odds ratio effect size estimates for binary traits i.e. hypertension (HTN); SE, standard error.
P: P-values (two-tailed) using Bolt-LMM v2.3.2 [69], with p<5E-4 in bold and p<5E-08 highlighted in green.

Supplementary Data 4: Stage 1 Discovery GWAS genomic regions, associated with five BP traits at p<5E-06.

Suggestive associated genomic regions (or loci) (p<5E-06) from the Stage 1 discovery GWAS (identified in FUMA).
Abbreviations: CHR, chromosomes; POS, gene position, SNP, single nucleotide polymorphism; EA, effect allele; RA, reference allele; EAF, effect allele frequency; β, Beta i.e. effect size estimates for continuous trait i.e. systolic blood pressure (SBP), diastolic blood pressure (DBP), pulse pressure (PP) and mean-arterial pressure (MAP); OR, odds ratio effect size estimates for binary traits i.e. hypertension (HTN); SE, standard error.
Position is given for Build 37 (GRCh37/hg19). Duplicate SNPs in bold. P-value<5E-8 in bold.
* Nearest annotated gene(s) given.
Beta, SE: EAF and P-value (two-tailed) calculated using RE2 (Han and Eskin's random-effects) model implemented in METASOFT v2.0.1 [70].
P-value, Stage 1 meta-analysis P-value for RE2.
Beta and SE for Random Effects (RE) as provided by METASOFT v2.0.1 [70].
Start: Window Start of the locus defined by FUMA [74]; End: Window End of the locus as defined by FUMA [74].
* Red Text=SNP name found by chr:pos.
* Red Highlight=Duplicate SNPs.

Supplementary Data 5: Stage 2 Discovery GWAS genomic regions, associated with five BP traits at p<5E-06.

Suggestive associated genomic regions (or loci) (p<5E-06) from the Stage 2 meta-analysis GWAS (identified in FUMA).
Abbreviations: CHR, chromosomes; POS, gene position, SNP, single nucleotide polymorphism; EA, effect allele; RA, reference allele; EAF, effect allele frequency; β, Beta i.e. effect size estimates for continuous trait i.e. systolic blood pressure (SBP), diastolic blood pressure (DBP), pulse pressure (PP) and mean-arterial pressure (MAP); OR, odds ratio effect size estimates for binary traits i.e. hypertension (HTN); SE, standard error.
Position is given for Build 37 (GRCh37/hg19). Duplicate SNPs in bold. P-value<5E-8 in bold.
* Nearest annotated gene(s) given.
Beta, SE: EAF and P-value (two-tailed) calculated using RE2 (Han and Eskin's random-effects) model implemented in METASOFT v2.0.1 [70].
P-value, Stage 1 meta-analysis P-value for RE2.
Beta and SE for Random Effects (RE) as provided by METASOFT v2.0.1 [70].
Start : Window Start of the locus defined by FUMA [74]; End : Window End of the locus as defined by FUMA [74].

Supplementary Data 6: Stage 1 Discovery GWAS SNPs, associated with five BP traits at p<5E-06 (based on the AWI-Gen cohort regions).

Stage 1 meta-analysis of AWI-GEN regions i.e. East (E), South (S) and West (W) African subsets.
Given for Build 37 (GRCh37/hg19).
Arranged in order of trait, chromosome, position and SNP. 437 SNPs with duplicate SNPs in bold.
Abbreviations: CHR, chromosomes; POS, gene position; SNP, single nucleotide polymorphism; A1, effect allele; A2, reference allele; AF, effect allele frequency; FE, fixed-effects; RE2, Han and Eskin's random-effects; Beta, effect size RE estimates for continuous trait i.e. systolic blood pressure (SBP), diastolic blood pressure (DBP), pulse pressure (PP) and mean-arterial pressure (MAP); OR, odds ratio effect size estimates for binary traits i.e. hypertension (HTN); SE, standard error; Het; heterozygosity; I2, variation percentage across studies due to heterogeneity.
Beta, SE: EAF and P-value (two-tailed) calculated using RE2 (Han and Eskin's random-effects) model implemented in METASOFT v2.0.1 [70].
P: P-values RE2 for P-value<5E-6, with each regions P-value<5E-4 in bold.
Beta and SE for Random Effects (RE) as provided by METASOFT v2.0.1 [70].
Previous Studies AF: Extracted from the PhenoScanner database, which uses the AF of the first allele in 1000G phase 3, for each ancestry [37].
Ancestries: Afr, African; Amr, Admixed American; Eas, East Asian; Eur, European; Aas, African Americans.

Supplementary Data 7: Stage 2 Discovery GWAS SNPs, associated with five BP traits at p<5E-06 (based on Stage 1, UGR and UKBB datasets).

Stage 2 meta-analysis of Stage 1 (S1) with UKBBa and UGR subsets.
Given for Build 37 (GRCh37/hg19).
Arranged in order of trait, chromosome, position and SNP. 437 SNPs with duplicate SNPs in bold.
Abbreviations: CHR, chromosomes; POS, gene position; SNP, single nucleotide polymorphism; A1, effect allele; A2, reference allele; AF, effect allele frequency; FE, fixed-effects; RE2, Han and Eskin's random-effects; Beta, effect size RE estimates for continuous trait i.e. systolic blood pressure (SBP), diastolic blood pressure (DBP), pulse pressure (PP) and mean-arterial pressure (MAP); OR, odds ratio effect size estimates for binary traits i.e. hypertension (HTN); SE, standard error; Het; heterozygosity; I2, variation percentage across studies due to heterogeneity.
Beta, SE: EAF and P-value (two-tailed) calculated using RE2 (Han and Eskin's random-effects) model implemented in METASOFT v2.0.1 [70].
P: P-values RE2 for P-value<5E-6, with each regions P-value<5E-4 in bold.
Beta and SE for Random Effects (RE) as provided by METASOFT v2.0.1 [70].
Previous Studies AF: Extracted from the PhenoScanner database, which uses the AF of the first allele in 1000G phase 3, for each ancestry [37].
Ancestries: Afr, African; Amr, Admixed American; Eas, East Asian; Eur, European; Aas, African Americans.

Supplementary Data 8: Exact replication of previous studies (p<5E-08) with the Stage 1 and Stage 2 GWAS (p<5E-04).

Ranked by chromosome and position for SBP, DBP, HTN, PP and MAP for Stages 1 and 2 for 592 replicated SNPs.
Abbreviations: CHR, chromosomes; POS, gene position; SNP, single nucleotide polymorphism; EA, effect allele; RA, reference allele; EAF, effect allele frequency with minor allele frequencies (MAF) reported for previous studies; Beta, effect size estimates for continuous trait i.e. systolic blood pressure (SBP), diastolic blood pressure (DBP), pulse pressure (PP) and mean-arterial pressure (MAP); OR, odds ratio effect size estimates for binary traits i.e. hypertension (HTN); SE, standard error; PMID, PubMed ID.
Beta, SE: EAF and P-value (two-tailed) calculated using RE2 (Han and Eskin's random-effects) model implemented in METASOFT v2.0.1 [70].
P: P-values RE2 for P-value<5E-04 (stage 1 and 2) against P-value<5E-08 in previous studies.
Beta and SE for Random Effects (RE) as provided by METASOFT v2.0.1 [70].
Previous Studies AF: Extracted from the PhenoScanner database, which uses the AF of the first allele in 1000G phase 3, for each ancestry [37].
Ancestries: Aas, African Americans; Aca, Afro-Caribbean; Afr, African; Amr, Admixed American; Asi, Asian; Bri, British; Eas, East Asian; Eur, European; Han, Han Chinese; His, Hispanic; Kuw, Kuwaiti; Lat, Latino; Nat; Native American; Nig, Nigerian; NR; Not reported; Sas, South Asian.
Ancestry Groups: (1) African only (African): Aas, Nig. (2) Multi ancestry with African (MulA): MulA1 (Aas, His/Lat, Asi, Nat, Eur, Lat, Aas, Eas, Asi), MulA2 (Eur, Aas, His); MulA3 (Eur, Aas/Aca, Asi, His/Lat Amr); MulA4 (Eur, Afri,

His, Asi, Nat); MulA5 (Eur, Lat, Aas, Afr Bri, Eas, Asi, mixed/unknown); MulA6 (Eur, Lat, Aas, Eas, Asi). (3) Multi ancestry without African (Multi): Multi1(Eur, Asi); Multi2 (Eur, Eas).

Supplementary Data 9: GWAS Catalog traits for SNPs in Region for BP-related traits with GW significance (p<5E-08).

Positions shown for GW significant associations i.e. systolic blood pressure (SBP) around the *P2RY1* region (rs77846204, p=4.95E-08) and pulse pressure (PP) around the *LINC01256* region (rs115808348, p=1.76E-08, intergenic *ELL2P2* – also consisting of rs62317311 (p=8.92E-07), for the AWI-Gen Stage 1 GWAS (N=10,775).
Abbreviations: CHR, chromosomes; POS, gene position; SNP, single nucleotide polymorphism; EA, effect allele; RA, reference allele; EAF, effect allele frequency with minor allele frequencies (MAF) reported for previous studies; Beta, effect size estimates for continuous trait i.e. SBP and PP; SE, standard error; PMID, PubMed ID; P: P-value (two-tailed).
Using the Locuszoom workflow showing GW association with (a) SBP and (b) PP for the AWI-Gen Stage 1 GWAS (N=10,775).
Flanking regions: (a) SBP(rs77846204, p=4.95E-08) around the *P2RY1* region (chr3:151582544-153582544[1MB]) and PP(rs115808348, p=1.76E-08) around the *LINC01256* region (chr4:132259109-134259109[1MB]).*GW Trait position within range.
Bold=Around GW trait.

Supplementary Data 10: Fine-mapping the region around independent significant SNPs for BP-related traits with GW significance (p<5E-08).

Positions shown for GW significant associations i.e. systolic blood pressure (SBP) around the *P2RY1* region (rs77846204, p=4.95E-08) and pulse pressure (PP) around the *LINC01256* region (rs115808348, p=1.76E-08, intergenic *ELL2P2* – also consisting of rs62317311 (p=8.92E-07), for the AWI-Gen Stage 1 GWAS (N=10,775).
Abbreviations: CHR, chromosomes; POS, gene position; SNP, single nucleotide polymorphism; EA, effect allele; RA, reference allele; EAF, effect allele frequency with minor allele frequencies (MAF) reported for previous studies; Beta, effect size estimates for continuous trait i.e. SBP and PP; SE, standard error; P: P-value (two-tailed).
The log10 Bayes factors (log20bf)>2 are highlighted in green.

Supplementary Data 11: Functional annotation of genetic positions with GW significance (p<5E-08) for SBP (rs77846204) and PP (rs115808349).

Positions shown for GW significant associations i.e. systolic blood pressure (SBP) around the *P2RY1* region (rs77846204, p=4.95E-08) and pulse pressure (PP) around the *LINC01256* region (rs115808348, p=1.76E-08, intergenic *ELL2P2* – also consisting of rs62317311 (p=8.92E-07), for the AWI-Gen Stage 1 GWAS (N=10,775).
Abbreviations: CHR, chromosomes; POS, gene position; SNP, single nucleotide polymorphism; EA, effect allele; RA, reference allele; EAF, effect allele frequency with minor allele frequencies (MAF) reported for previous studies; Beta, effect size estimates for continuous trait i.e. SBP and PP; SE, standard error; P: P-value (two-tailed).

Supplementary Data 12: Functional mapping of genetic positions with GW significance (p<5E-08) for SBP (rs77846204) and PP (rs115808349).

Positions shown for GW significant associations i.e. systolic blood pressure (SBP) around the *P2RY1* region (rs77846204, p=4.95E-08) and pulse pressure (PP) around the *LINC01256* region (rs115808348, p=1.76E-08, intergenic *ELL2P2* – also consisting of rs62317311 (p=8.92E-07), for the AWI-Gen Stage 1 GWAS (N=10,775).
P: P-value (two-tailed) using FUMA [74].

Supplementary Data 13: Predictivity of PRS models in the AWI-Gen cohort.

Performance and reclassification of a PRS model for the risk prediction was estimated using AUROC and AUC metrics were conducted using the pROC [79] package in R [72]. GWASs (discovery) were applied to the AWI-Gen cohort (target, N=10,676) for systolic blood pressure (SBP) and diastolic blood pressure (DBP): (1) African: Two African-ancestry cohorts i.e. UKBBa (n=3,058) and UGR (n=6,400) [45] (2) European: UK biobank and ICBP (N=757,601) [16,46] and (3) Multi-ancestry: PAGE (N=49,839 with 17,152 African-ancestry) cohort [40,46].
P: P-value (two-tailed) were estimated using PRSice-2 V2.3.5 [78]; PT, P-value threshold.
R2, variance explained; Adj, R2, adjusted variance explained; VEP, Variance explained by pheno.
AUC lower bound>0.5 suggests significance at a 95% confidence interval, highlighted in green.
