# Supplementary material for: Genome-wide association study meta-analysis of blood pressure traits and hypertension in sub-Saharan African populations: an AWI-Gen study
Source: Nat Commun. 2023 Dec 16;14:8376. doi: 10.1038/s41467-023-44079-0 (PMC10725455; doi:10.1038/s41467-023-44079-0)
Supplement: Supplementary file 5 — Reporting Summary [file 41467_2023_44079_MOESM5_ESM.pdf]

nature portfolio

Corresponding author(s): Surina Singh, Michele Ramsay

Last updated by author(s): Nov 7, 2023

Reporting Summary

Nature Portfolio wishes to improve the reproducibility of the work that we publish. This form provides structure for consistency and transparency in reporting. For further information on Nature Portfolio policies, see our Editorial Policies and the Editorial Policy Checklist.

Statistics

For all statistical analyses, confirm that the following items are present in the figure legend, table legend, main text, or Methods section.

n/a	Confirmed	
☐	☒	The exact sample size (*n*) for each experimental group/condition, given as a discrete number and unit of measurement
☐	☒	A statement on whether measurements were taken from distinct samples or whether the same sample was measured repeatedly
☐	☒	The statistical test(s) used AND whether they are one- or two-sided<br>*Only common tests should be described solely by name; describe more complex techniques in the Methods section.*
☐	☒	A description of all covariates tested
☐	☒	A description of any assumptions or corrections, such as tests of normality and adjustment for multiple comparisons
☐	☒	A full description of the statistical parameters including central tendency (e.g. means) or other basic estimates (e.g. regression coefficient) AND variation (e.g. standard deviation) or associated estimates of uncertainty (e.g. confidence intervals)
☐	☒	For null hypothesis testing, the test statistic (e.g. *F*, *t*, *r*) with confidence intervals, effect sizes, degrees of freedom and *P* value noted<br>*Give P values as exact values whenever suitable.*
☒	☐	For Bayesian analysis, information on the choice of priors and Markov chain Monte Carlo settings
☒	☐	For hierarchical and complex designs, identification of the appropriate level for tests and full reporting of outcomes
☐	☒	Estimates of effect sizes (e.g. Cohen's *d*, Pearson's *r*), indicating how they were calculated

Our web collection on statistics for biologists contains articles on many of the points above.

Software and code

Policy information about availability of computer code

Data collection	N/A<br>No software was used for data collection.
Data analysis	The H3ABioNet/H3Agwas GWAS pipeline workflow was employed for QC, association testing, meta-analysis and fine-mapping (https://github.com/h3abionet/h3agwas). Sanger Imputation Server (https://imputation.sanger.ac.uk), 2.3M SNP H3Africa genotyping array (https://chipinfo.h3abionet.org), STATA V15, R (qqman, pROC), Quanto V1.2.3, BOLT-LMM V2.3.2, METASOFT V2.0.1, FUMA pipeline (https://fuma.ctglab.nl), PRSice-2 V2.3.5, FINEMAP V1.0, Locuszoom (https://my.locuszoom.org/).

For manuscripts utilizing custom algorithms or software that are central to the research but not yet described in published literature, software must be made available to editors and reviewers. We strongly encourage code deposition in a community repository (e.g. GitHub). See the Nature Portfolio guidelines for submitting code & software for further information.

Data

Policy information about availability of data

All manuscripts must include a data availability statement. This statement should provide the following information, where applicable:

- Accession codes, unique identifiers, or web links for publicly available datasets
- A description of any restrictions on data availability
- For clinical datasets or third party data, please ensure that the statement adheres to our policy

The AWI-Gen data set is available from the European Genome-phenome Archive (EGA) database (https://ega-archive.org/), with accession number EGAS00001002482 (phenotype dataset: EGAD00001006425; genotype dataset: EGAD00010001996, genome assembly: GRCh37/hg19). The availability of these datasets are subject to controlled access through, the Data and Biospecimen Access Committee of the H3Africa Consortium. The processed data generated in this study are provided in Supplementary Material. The summary statistics reported in the paper are accessible on the GWAS Catalog (https://www.ebi.ac.uk/gwas/). Permission was obtained to access the genotype and phenotype dataset for UKBB (research project number: 63215) (as described in Methods).
Publicly available databases include (1) GWAS Catalog 17 (https://www.ebi.ac.uk/gwas/; BP, EFO_0004325; SBP, EFO_0006335; DBP, EFO_0006336; HTN, EFO_0000537; PP, EFO_0005763; MAP: EFO_0006340), (2) PhenoScanner 37 (http://www.phenoscanner.medschl.cam.ac.uk/, Traits: BP, SBP, DBP, HTN, PP, MAP). Other summary statistics reported in the paper are accessible on the GWAS Catalog (https://www.ebi.ac.uk/gwas/) for (1) Gurdasani, et al. 45 African-ancestry UGR cohort (SBP: GCST009053, DBP: GCST009052). (2) Evangelou, et al. 16 European-ancestry UKBB & ICBP cohorts (SBP: GCST006624, DBP: GCST006630, PP: GCST006629). (3) Wojcik, et al. 46 multi-ancestry PAGE cohort (SBP: GCST008044, DBP: GCST008029). Source data are provided as a Source Data file.

Research involving human participants, their data, or biological material

Policy information about studies with human participants or human data. See also policy information about sex, gender (identity/presentation), and sexual orientation and race, ethnicity and racism.

Reporting on sex and gender	Term used: "Sex"<br>Sex data has been collected, with only number of individuals / percentages reported (Table). Sex was used as a covariates for association and prediction analyses.
Reporting on race, ethnicity, or other socially relevant groupings	Term used: "Ancestry"<br>Ancestry was used to distinguish between different ethnicities. This studies African-ancestry analyses were compared with other studies ethnic groups i.e. African-ancestry, European-ancestry and multi-ancestry.
Population characteristics	Participants were from the AWI-Gen study (10,775 participants, with the majority (89.3%) aged between 40-60 years), located in three African regions (East, West, South) from six sites within four countries i.e. East - Kenya (Nairo DNAbi); West - Burkina Faso (Nanoro) and Ghana (Navrongo); and South - South Africa (Agincourt, Dikgale, Soweto). A digital sphygmomanometer (Omron M6, Omron, Kyoto, Japan) was used for BP measurements, following the Seventh Report of the Joint National Committee on Prevention, Detection, Evaluation, and Treatment of High BP (JNC7) guidelines. Sociodemographic data was captured. Whole blood samples were collected for DNA extraction and analysis.
Recruitment	The AWI-Gen study collected DNA and bio markers from about 12,000 participants across 6 centers in 4 African countries. Genotype data of ~11,000 samples was generated on the 2.3M SNP H3Africa genotyping array designed to include common African variants (https://chipinfo.h3abionet.org). Exclusion criteria for the study were: pregnant women, close relatives of existing participants (first and second-degree relatives), recent immigrants (who migrated <10 years ago into the region) and individuals with physical impairments preventing measurement of BP. Further study cohort details can be found in Ramsay, et al. 2016 and Ali, et al. 2018. Singh, et al. 2021 was used as a reference to identify previous GWAS for BP related traits in African populations, identifying only one study with summary statistics for SBP and DBP in a continental African population i.e. Gurdasani, et al. 2019.
Ethics oversight	Ethical approval was obtained from the Human Research Ethics Committee (HREC) (Medical) of the University of the Witwatersrand (Protocol Number: M190927). This was a sub-study to the AWI-Gen study (Protocol Numbers: M121029, M170880, M2210108). Each of the participating sites also obtained ethics approval from their respective ethics committees. AWI-Gen sample data was used as permitted by the informed consent provided by the study participants and according to the H3Africa policies and guidelines (www.h3africa.org).

Note that full information on the approval of the study protocol must also be provided in the manuscript.

Field-specific reporting

Please select the one below that is the best fit for your research. If you are not sure, read the appropriate sections before making your selection.

☒ Life sciences ☐ Behavioural & social sciences ☐ Ecological, evolutionary & environmental sciences

For a reference copy of the document with all sections, see nature.com/documents/nr-reporting-summary-flat.pdf

Life sciences study design

All studies must disclose on these points even when the disclosure is negative.

Sample size	This was a cross-sectional study where sample size was determined by data availability.<br><br>Participants were from the AWI-Gen study (10,775 participants, with the majority (89.3%) aged between 40-60 years), located in three African regions (East, West, South) from six sites within four countries i.e. East - Kenya (Nairobi); West - Burkina Faso (Nanoro) and Ghana (Navrongo); and South - South Africa (Agincourt, Dikgale, Soweto). Singh, et al. 2021 was used as a reference to identify previous GWAS for BP related traits in African populations, identifying only one study with summary statistics for SBP and DBP in a continental African population i.e. Gurdasani, et al. 2019.<br><br>Stage 1 GWAS (N=10.8K) was conducted for all five BP-related traits, and a meta-analysis was performed using the sumary statistics of the GWAS for each AWI-Gen region (East, South, West). Stage 2 GWAS (N=20.3K) was conducted for SBP and DBP only, and was a meta-analysis of the Stage 1 results with other African-ancestry (n=9.5K) summary statistics i.e. UGR (n=6,400) and UKBBa (n=3,058).
Data exclusions	Exclusion criteria for the study were: pregnant women, close relatives of existing participants (first and second-degree relatives), recent immigrants (who migrated <10 years ago into the region) and individuals with physical impairments preventing measurement of BP.<br><br>The H3ABioNet/H3Agwas QC pipeline workflow (https://github.com/h3abionet/h3agwas/tree/master/qc) was used to conduct QC analysis for the AWI-Gen dataset, as previously described 3: (1) SNPs with high missingness (>0.02), low minor allele frequency (MAF) (<0.01), and extreme deviation from Hardy Weinberg Equilibrium proportions (HWE) (<0.0005) were excluded; (2) Samples with high genotype missingness (>0.01) and discordant sex information were removed; (3) Mitochondrial, Y and X chromosome SNPs, including SNPs that did not match the Genome Reference Consortium Human Build 37 (GRCh37/hg19) reference alleles were removed. The same steps were followed for the UKBBa dataset. The Winsorise very extreme value approach was used to assess outliers i.e. the values should be <6 standard deviations (SD) above or below the mean, but no such values were observed (Figure S 1).<br><br>Only participants with good quality phenotype and genotype data were used for the GWAS analyses (N=10,775). The genome assembly (base pair position) was the GRCh37/hg19.
Replication	Replication of the Stage 1 and 2 GWAS with previous studies, was assessed by comparing GW associations (p<5E-08) against SNPs with suggestive associations (p<5E-04). In addition, replication of GW signals found in previous studies (p<5E-08) were compared against the Stage 1 and 2 GWAS suggestive associations (p<5E-04). The H3ABioNet/H3Agwas Replication pipeline workflow was implemented to conduct replication analysis (https://github.com/h3abionet/h3agwas/tree/master/replication).<br><br>Several replicating known associations were identified.
Randomization	N/A<br>This study analyses four continuous BP-related traits (systolic BP (SBP), diastolic BP (DBP), pulse pressure (PP) and mean-arterial pressure (MAP)) and one categorical trait (HTN). and did not allocate any samples to groups. Therefore randomization is not relevant to our study design.
Blinding	N/A<br>The study does not involve any treatment or sharing of information with participants. Therefore blinding was not relevant to our study design.

Reporting for specific materials, systems and methods

We require information from authors about some types of materials, experimental systems and methods used in many studies. Here, indicate whether each material, system or method listed is relevant to your study. If you are not sure if a list item applies to your research, read the appropriate section before selecting a response.

Materials & experimental systems

n/a	Involved in the study	
☒	☐	Antibodies
☒	☐	Eukaryotic cell lines
☒	☐	Palaeontology and archaeology
☒	☐	Animals and other organisms
☒	☐	Clinical data
☒	☐	Dual use research of concern
☒	☐	Plants

Methods

n/a	Involved in the study	
☒	☐	ChIP-seq
☒	☐	Flow cytometry
☒	☐	MRI-based neuroimaging
